# Purification and Characterization of Two New Allergens from the Venom of *Vespa magnifica*

**DOI:** 10.1371/journal.pone.0031920

**Published:** 2012-02-27

**Authors:** Su An, Lingling Chen, Ji-Fu Wei, Xuening Yang, Dongying Ma, Xuemei Xu, Xueqing Xu, Shaoheng He, Jia Lu, Ren Lai

**Affiliations:** 1 Biotoxin Units of Key Laboratory of Animal Models and Human Disease Mechanisms, Kunming Institute of Zoology, Chinese Academy of Sciences, Kunming, Yunnan, China; 2 School of Life Sciences, University of Science and Technology of China, Hefei, Anhui, China; 3 Clinical Research Centre, the First Affiliated Hospital of Nanjing Medical University, Nanjing, Jiangsu, China; 4 Clinical Laboratory and the Otolaryngological Department, The First Affiliated Hospital of Kunming Medical College, Kunming, Yunnan, China; 5 Graduate School of the Chinese Academy of Sciences, Beijing, China; Natural Resources Canada, Canada

## Abstract

Due to poor diagnostic facilities and a lack of medical alertness, allergy to *Vespa* wasps may be underestimated. Few allergens have been identified from *Vespa* wasps.

Possible native allergen proteins were purified from the wasp venoms (WV) (*Vespa magnifica* Smith) by gel filtration, ion exchange chromatography, respectively. Their sequences were determined by Edman degradation and cDNA cloning. Their allergenicities were assayed by enzyme-linked immunosorbent assay inhibition tests (ELISA-IT), immunoblots, and skin prick tests (SPTs). Their cross allergencities with Tab y 2 and Tab y 5 purified from the horsefly (*Tabanus yao Macquart*) were also determined. Two native allergens were identified from the WV, respectively. They are a 25-KDa antigen 5 protein (Ag5) (Vesp ma 5) and a 35-KDa hyaluronidase (Vesp ma 2). They represented major allergens in *Vespa magnifica* by immunoblots and SPTs. ELISA inhibition of pooled sera IgE reactivity to both the WV and the horsefly salivary gland extracts (HSGE) using four purified allergens (Vesp ma 2, Vesp ma 5 and previously purified Tab y 2 and Tab y 5) was significant. Their cross allergenicities were confirmed by ELISA-IT, immunoblots, and SPTs. They represented the cross reactive allergens from wasp and horsefly and proved the so called wasp-horsefly syndrome.

## Introduction

Anaphylaxis from insect venom is mostly caused by Hymenoptera stings, including vespids of the genera *Vespula*, *Vespa*, *Dolichovespula*, and *Polistes* and by apids of the genera *Apis* and *Bombus*. Three ant genera are also of importance: *Solenopsis*, *Myrmecia* and *Pachycondyla* [1]–[5]. Many cases of wasp allergy with severe allergic reactions have been documented. The symptoms induced by wasp allergy include itch, urticaria, angioedema, bronchial constriction, shock, pharyngeal constriction, shortness of breath, unconsciousness, nausea, vomiting, shivers and profuse perspiration, which are similar to the allergic symptoms induced by stinging from other insects of the order of Hymenoptera.

There are more than 24 species in the genus of *Vespa* wasps but allergens are only found in the venoms of *Vespa crabro* [6], [7]. Due to poor diagnostic facilities and a lack of medical alertness, allergy to *Vespa* wasps may be underestimated. Few allergens have been identified from *Vespa* wasps. They are Vesp c 5 (Antigen 5) and Vesp c 1 (Phospholipase A_1_) [6], [7]. However, these two allergens' allergenicity is poorly understood. Furthermore, considering coexistent anaphylaxis to Diptera and Hymenoptera, concomitant sensitization to Hymenoptera venoms in subjects allergic to horseflies seems to be frequent (The wasp-horsefly syndrome) [8]–[10]. However, no cross-reactive allergens, which contribute to the coexistent anaphylaxis to *Vespa* wasp and horsefly, are known.

Many active compounds with anti-coagulation, anti-platelet, anti-inflammation, and immunosuppressant activities were isolated from the *Vespa* wasp, *Vespa magnifica* [11]–[15]. However, no allergens from the venom of *V. magnifica* have been purified and characterized. In the present study, we purified and characterized two novel allergens that we named Vesp ma 2 and Vesp ma 5 from the venom of *V. magnifica* and investigated their allergenicity.

## Materials and Methods

### Ethics Statement

The study protocol was approved by the ethics committee of the Institutional Review Board of the Kunming Institute of Zoology, Chinese Academy of Sciences.Written informed consent for the use of blood samples and skin test were obtained from all participants before study entry. We also obtained written informed consent from the next of kin, carers or guardians on the behalf of the minors/children participants involved in our study.

### Patient selection

Sera were obtained from 33 subjects with wasp allergy, 12 children age 6 to 18 years (mean 12.6 years) and 21 adults age 19–61 years (mean 41.2 years). They share similar allergic reactions including some of the symptoms of itch, urticaria, angioedema, bronchial constriction, shock, pharyngeal constriction, shortness of breath, unconsciousness, nausea, vomiting, shivers, and profuse perspiration. Twenty control sera were from individuals who had negative horsefly bite and wasp stinging tests. Sera were obtained from 37 subjects with horsefly allergy in our previous study [16]–[17], 17 children (46%) age 6 to 18 years (mean 12.1 years) and 20 adults (54%) age 19–59 years (mean 37.6 years), with immediate allergic reactions after the bites of *T. yao*. All sera were stored at −80°C.

### Collection of horsefly and SGE preparation

The procedures for the preparation of SGE (about 60 000, average weight 0.17 g) from 10 kg horseflies of *T. yao* Macquart and collection of horseflies were performed according to our previous reported method [16]–[17]. The salivary glands were excised and transferred into 0.1 M phosphate buffer solution, pH 6.0 (PBS), and homogenized in the same solution containing protease inhibitor cocktail and centrifuged at 5000 *g* for 10 min. The supernatant was termed SGE and lyophilized. 4.1 g total lyophilized SGE sample was obtained.

### *Vespa* wasp venom collection

Venoms of *V. magnifica* were collected according to our previous method [12], [18]. Adult *Vespa* wasps were collected and subjected to electronic stimulation (3–6 volts). Approximately 0.1 mg of venom can be obtained from one adult worker wasp. After electronic stimulation and venom collection, wasps were released. In total, 5 g of venom (wet weight) was obtained from about 50, 000 worker wasps.

### Allergen purification from *Vespa* wasp venoms

**Figure 1:**
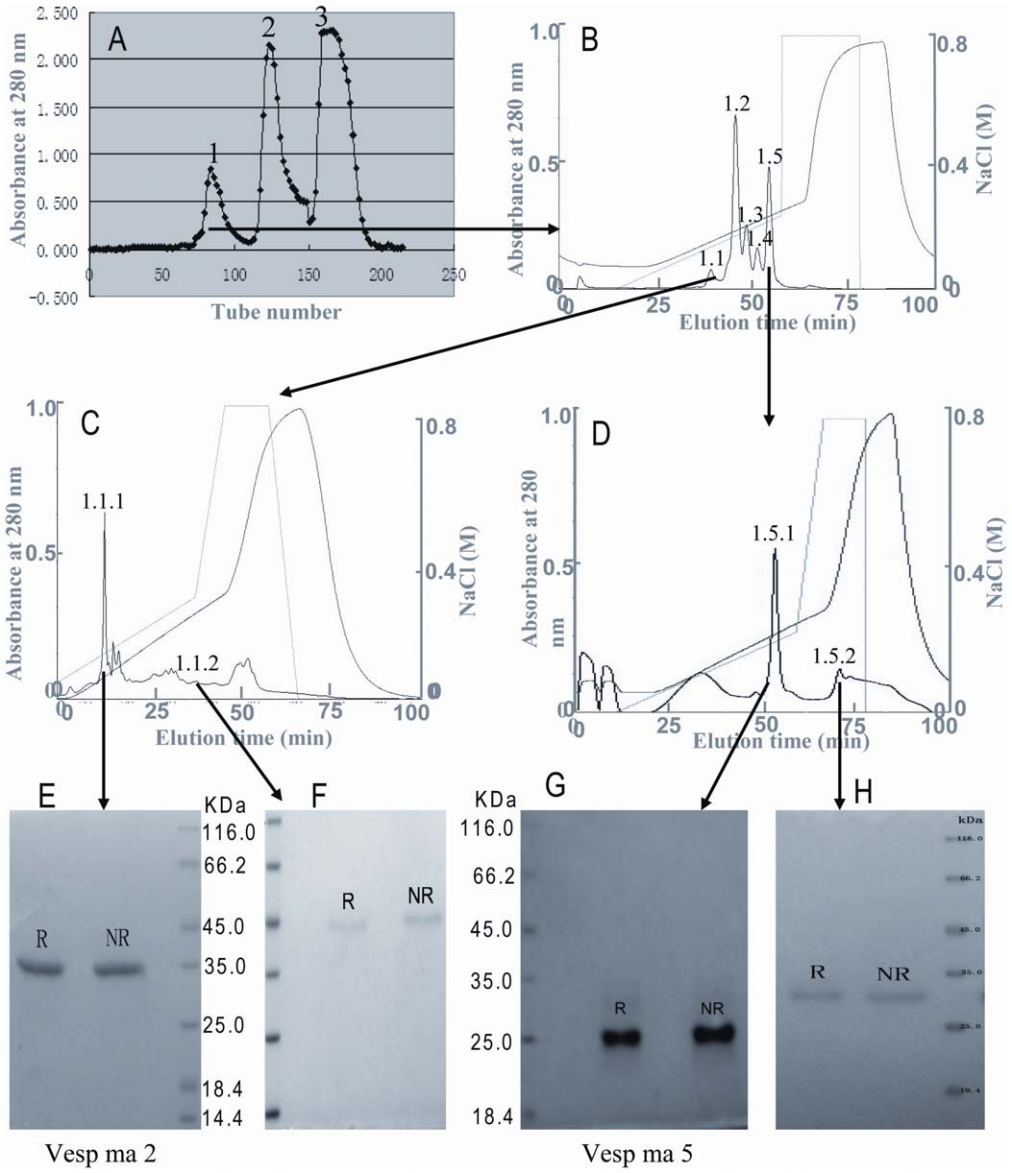
Purification of allergens from the wasp venoms (WV), *V. magnifica*. **A:** WV aliquot of 0.2 g was applied to a Sephadex G-75 gel filtration. The absorbance of the eluate was monitored at 280 nm. **B**: Fraction 1 from Sephadex G-75 gel filtration was subjected to AKTA Resource S cationic exchange chromatography. The elution was performed at a flow rate of 1 ml/min with the indicated NaCl gradient. **C**: The first protein peak after Resource S cationic exchange chromatography (peak 1.1) was subjected to AKTA Mono S cationic exchange chromatography. The elution was performed at a flow rate of 1 ml/min with the indicated NaCl gradient. **D**: The fifth protein peak after Resource S cationic exchange chromatography (peak 1.5) was subjected to AKTA Mono S cationic exchange chromatography. The elution was performed at a flow rate of 1 ml/min with the indicated NaCl gradient. **E & F**: SDS-PAGE analysis of purified allergens of Vesp ma 5 and Vesp ma 2 in 15% gel concentration. R: reduced; NR: non-reduced.

Aliquots of *Vespa* wasp venoms (WV, 0.2 g) dissolved in 6 ml 0.1 M PBS, pH 6.0 were applied to a Sephadex G-75 (Superfine; Amersham Biosciences; 2.6×100 cm) gel filtration column and eluted with the same buffer (Fig. 1A). Each fraction was subjected to ELISA inhibition testing as described below. The eluted protein peaks, which show ELISA inhibition activities were pooled and purified further by cationic exchange columns of Resource S (10 ml volume, Amersham Biosciences) and Mono S (1 ml volume, Amersham Biosciences) as illustrated in Fig. 1B–F. The purities of purified proteins were determined by SDS-PAGE. The protein concentration was determined by a protein assay kit (Bio-Rad, Hercules, CA) with BSA as a standard.

### Structural analysis

The amino acid sequences of the N-terminus and partial internal amino acid fragments recovered from the trypsin hydrolysis were determined by automated Edman degradation on an Applied Biosystems pulsed liquid-phase sequencer, model 491.

### cDNA library construction and cDNA cloning

A cDNA library of the venom gland of *V. magnifica* was constructed as previous described [19], [20]. A PCR-based method for high stringency screening of DNA libraries was used for screening and isolating the clones. Specific primers in the sense direction were designed according to the peptide sequences determined by Edman degradation (Vesp ma 5: 5′-AA(T/C)AA(T/C)TA(T/C)TG(T/C)AG(A/G)AT(A/T/C) AA(A/G)TG-3′; Vesp ma 2: 5′-TC(A/T/C/G)GA(A/G)AG(A/G)CC(A/G/C/T)AA(A/G)AG(A/G)GT(A/G/C/T) TT-3′) and primer II A (5′-AAGCAGTGGTATCA ACGCAGAGT-3′) in the antisense direction provided by the SMART™ PCR cDNA synthesis kit were used in PCR reactions. DNA sequencing was performed on an Applied Biosystems DNA sequencer, model ABI PRISM 377.

### Hyaluronidase assay

Hyaluronidase assays were performed for the detection of hydrolysis of hyaluronic acid according to the method described by our previous work. [16] Tested samples with different concentrations were incubated with 30 µg/ml hyaluronic acid in 50 µl 10 mM Hepes buffer, pH 7.4 containing 0.15 M NaCl. Reactions were stopped by adding 50 µl of freshly prepared 2.5% cetylpyridinium chloride in 2% NaOH. The absorbance at 405 nm was measured after 5 min.

### Immunoblot analysis with sera from patients with horsefly or *Vespa* wasp allergy

Immunoblots for the detection of specific IgE binding were performed with purified Vesp ma 2 and Vesp ma 5 or horsefly allergens (Tab y 2 and Tab y 5), which were purified from *T. yao* Macquart [16], in allergic patients and 2 negative controls. Purified allergens (3 µg) were loaded on an SDS-PAGE with a gel concentration of 15% under reducing conditions and then transferred to nitrocellulose membranes. Membranes were incubated with sera from patients (1∶5 to 1∶20 in PBS-Tween [1% BSA, 10% normal goat serum]) for 90 min. After rinsing with PBS, the membranes were incubated with monoclonal anti-human IgE (peroxidase-labeled affinity purified antibody to Human IgE, KPL, Inc. Maryland). Color was developed by incubation of the membranes with substrate.

### Enzyme-linked immunosorbent assay (ELISA)

96-well plates were coated overnight at 4°C with 100 µl per well of 20 µg/ml SGE or WV in carbonate-bicarbonate buffer (0.05 mol/l, pH 9.6). Human serum samples (1∶50 dilution by 2% BSA, 0.05% Tween phosphate buffer saline) were pre-incubated with different concentrations of purified allergens for 1 hour at 37°C, and added to the plates for 2 hours at room temperature. Incubation of the diluted serum with saline was used as a positive control. After IgE binding, plates were incubated with horseradish peroxidase-labeled goat antihuman IgE (KPL, Inc. Maryland, 1∶2500), developed with tetramethylbenzidine peroxidase substrate (KPL). The absorbance at 650 nm was monitored on a microplate reader (Epoch Etock, BioTek).

### Skin Prick Tests

For the skin prick testing, no patients were taking medications (antihistamines, steroids, and other drugs) for at least 2 weeks. They have no dermatographia or active skin disorders. Skin prick tests were performed according to the standard procedure. The skin prick reaction was read at 15 min. Wheal sizes were expressed as the mean of the longest diameter and midpoint perpendicular diameter. A skin prick test result was considered positive if the mean wheal diameter was >3 mm.

## Results

### Purification of *Vespa* wasp allergens

As illustrated in Fig. 1A, the *Vespa* wasp venom was divided into three fractions by Sephadex G-75 gel filtration. Fraction 1 was found to contain binding activity with IgE from patient sera. Fraction 1 was subjected to AKTA Resource S cationic exchange as illustrated in Fig. 1B. Five protein fractions were eluted out as indicated 1.1 to 1.5 in Fig. 1B. Fraction 1.1 was found to contain hyaluronidase activity and subjected to further purification by Mono S cationic exchange as illustrated in Fig. 1C. Fraction 1.1.1 was the purified 35-KDa hyaluronidase (Fig. 1E, Fig. S1). Fraction 1.5 in Fig. 1B was also purified by Mono S cationic exchange as illustrated in Fig. 1D. Fraction 1.5.1 was the purified 25-KDa Ag5 (Fig. 1F). The IUIS Allergen Nomenclature Sub-Committee (www.allergen.org) has assigned to this purified allergens, antigen 5 and hyaluronidase, the following official numbers: Vesp ma 5 and Vesp ma 2, respectively.

### cDNA cloning of *Vespa* wasp allergens

**Figure 2:**
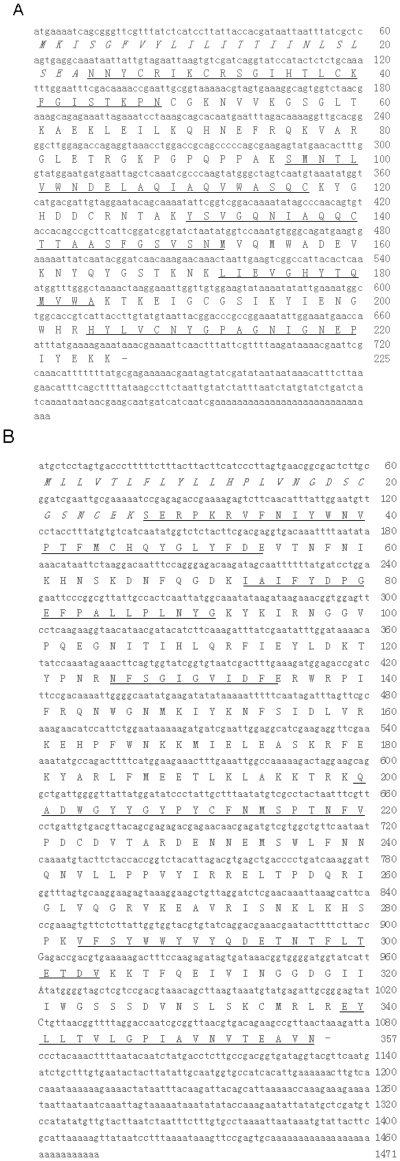
cDNA encoding the Vesp ma 5 (A) and Vesp ma 2 (B) from the wasp venom of *V. magnifica* and deduced amino acid sequence. The amino acid sequences of peptide fragments determined by Edman degradation are underlined. The predicted signal peptide is in italic. -: stop codon.

N-terminus and partial internal peptide fragments recovered from trypsin hydrolysis of purified Vesp ma 2 and Vesp ma 5 are illustrated in Fig. 2. Complete cDNA sequences encoding Vesp ma 5 and Vesp ma 2 were cloned from the wasp venom gland cDNA library. The GenBank accession numbers of antigen 5 and hyaluronidase were FR774918 and FR749885, respectively. The proprotein of Vesp ma 5 is composed of 225 amino acid residues (aa) including a predicted signal peptide (23 aa) and the mature Vesp ma 5 (202 aa) (Fig. 2A). This protein was found to contain the conserved SCP domain (Sc7 family of extracellular domains) found in insect Ag5 proteins.

The proprotein of Vesp ma 2 is composed of 357 aa including a predicted signal peptides (26 aa) and the mature Vesp ma 2 (331 aa) (Fig. 2B). As illustrated in Fig. S1, purified Vesp ma 2 showed strong hydrolytic activity on hyaluronic acid in a time-dependent manner.

### IgE Immunoblot

**Figure 3:**
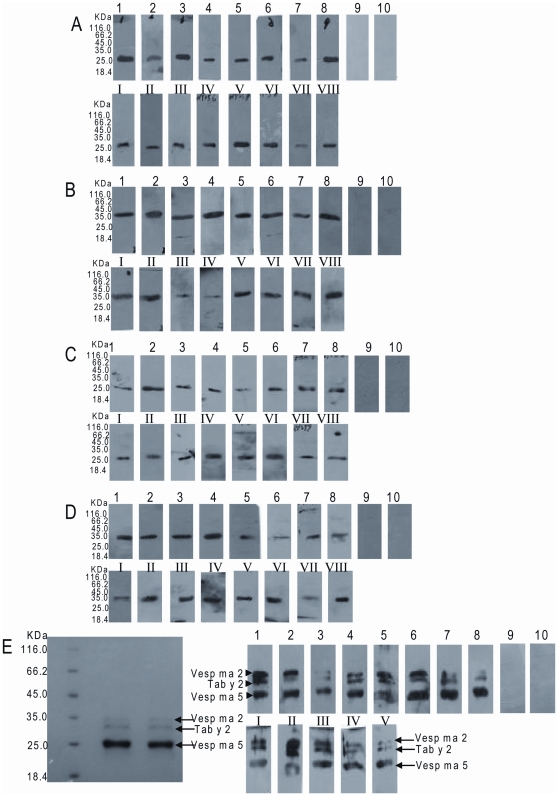
Immunoblot of IgE reactivity to Vesp ma 5(A), Vesp ma 2(B), Tab y 5(C), and Tab y 2(D). *Lanes* 1–8, representatives of subject serum from patients with wasp allergy; *Lanes* I–VIII, representatives of subject serum from patients with horsefly allergy; *Lanes* 9 and 10, negative controls. **E**: Three allergens (Vesp ma 5, Vesp ma 2, Tab y 2) were mixed and loaded on the same lane of SDS-PAGE (*Left*), and transferred to a nitrocellulose membrane for immunoblotting (*Right*). Comassie blue staining was performed to visualize proteins after PAGE-SDS. *Note*: Tab y 5 was not mixed with Vesp ma 5, Vesp ma 2, and Tab y 2 because it has the same molecular weight with Vesp ma 5.

Thirty and 31 of 33 (91.0% and 93.9%) of subjects' sera with *Vespa* wasp allergy reacted to Vesp ma 5 and Vesp ma 2, respectively, as measured by the immunoblotting technique. IgE immunoblots of a representative group of 8 patients and two controls are presented in Fig. 3A–D. IgE binding was detected in 30 and 31 of 37 (81.1% and 83.8%) of subjects' sera with horsefly allergy using Vesp ma 5 and Vesp ma 2, respectively. Our previous work showed that Tab y 5 and Tab y 2 reacted with 32 and 34 of 37 (86.5% and 91.8%) of subjects' sera presenting horsefly allergy, respectively. On the other hand, 27 and 26 of 33 (81.8% and 78.8%) of subjects' sera with wasp allergy reacted to Tab y 5 and Tab y 2, respectively, as assessed by IgE binding. Fig. 3E shows that IgE binding was positive to three proteins around 35 KDa, 34 KDa, and 25 KDa in patient sera. These masses correspond to the molecular weight of Vesp ma 2, Tab y 5, and Tab y 2, and were completely absent in all control subjects, when these allergens were loaded on the same lane.

### ELISA inhibition

**Figure 4:**
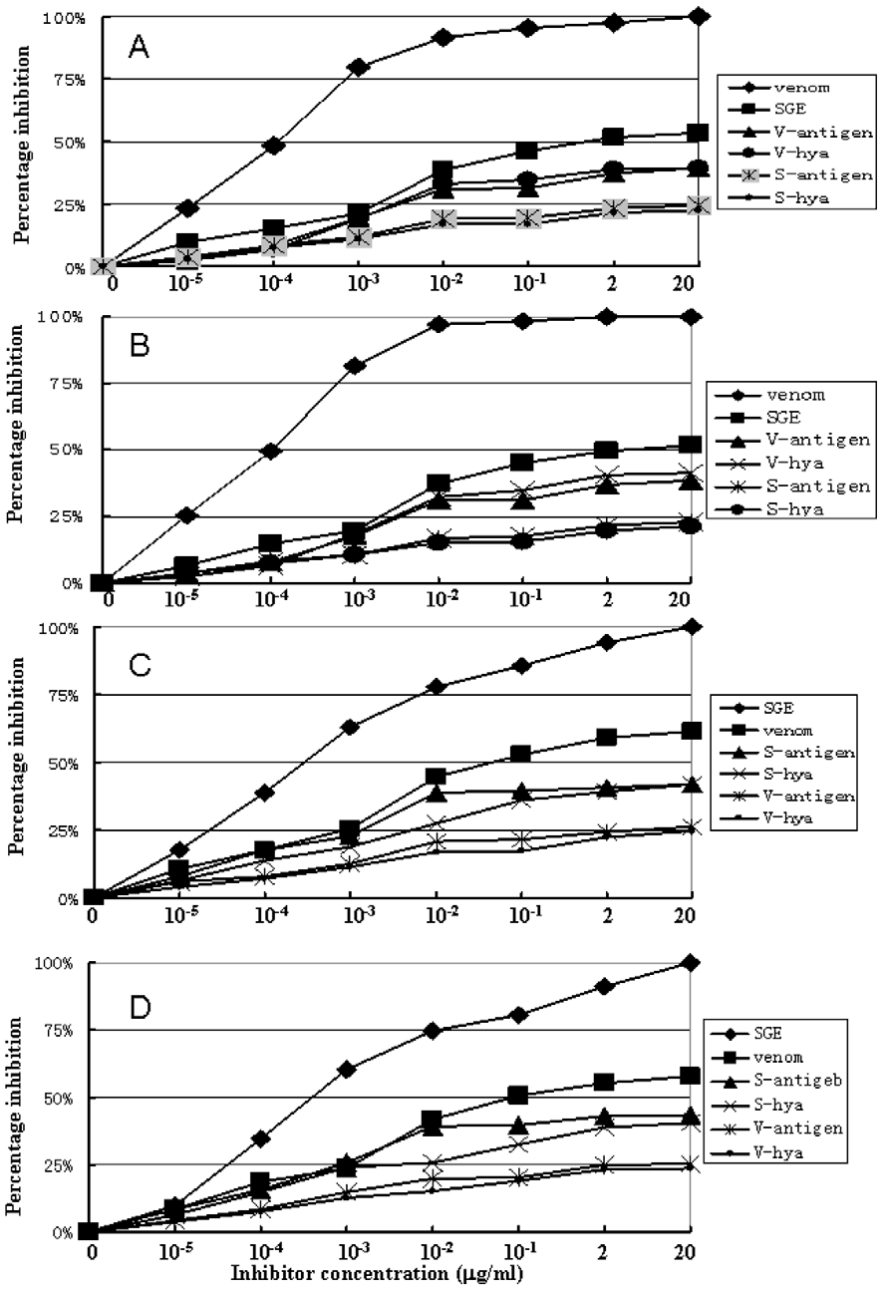
Cross ELISA inhibition of purified wasp and horsefly allergens with patients' serum from subject 2, 3 and 8. **A& B**: 20 µg/ml coated wasp venom's binding with serum IgE from subject 2 (A) and 8 (B) was inhibited by wasp venom, horsefly SGE, Vesp ma 5, Vesp ma 2, Tab y 5, and Tab y 2, respectively. **C& D**: 20 µg/ml coated horsefly SGE's binding with serum IgE from subject 3 (C) and 8 (D) was inhibited by wasp venom, horsefly SGE, Vesp ma 5, Vesp ma 2, Tab y 5, and Tab y 2, respectively. Inhibitor concentrations ranged from 10^−5^ µg to 20 µg/ml. SGE: salivary gland extracts; V-antigen: Vesp ma 5; V-hya: Vesp ma 2; S-antigen: Tab y 5; S-hya: Tab y 2.

For ELISA inhibition assays, the sera of three subjects (2, 3, and 8) which recognized Vesp ma 5, Vesp ma 2, Tab y 5, and Tab y 2 by immunoblot were chosen. All of these allergens could inhibit the binding of the subjects' IgE antibodies to the coated SGE and WV. Four representative ELISA inhibitions are illustrated in Fig. 4A–D. For example, the coated WV's binding to the subjects' IgE antibodies was inhibited by the WV, the SGE, Vesp ma 5, Vesp ma 2, Tab y 5, and Tab y 2 in a dose-dependent manner (Fig. 4A and B). Twenty µg/ml of WV could completely inhibit IgE binding. The maximal inhibition by the SGE, Vesp ma 5, Vesp ma 2, Tab y 5, and Tab y 2 was about 53.3, 39.5, 39.4, 24.6, and 22.6%, respectively. Two wasp allergens (Vesp ma 5 and Vesp ma 2) had stronger inhibitory ability to IgE binding with wasp venom than these horsefly allergens (Tab y 5 and Tab y 2). In the same situation, the coated SGE's binding to the subjects' IgE antibodies was also inhibited by the WV, the SGE, Vesp ma 5, Vesp ma 2, Tab y 5, and Tab y 2 in a dose-dependent manner (Fig. 4C and D). 20 µg/ml of the SGE could achieve 100% of the IgE inhibition whereas maximal inhibition by the WV, Vesp ma 5, Vesp ma 2, Tab y 5, and Tab y 2 was about 57.9, 25.3, 23.8, 43.6, and 40.6%, respectively. Two horsefly allergens (Tab y 5 and Tab y 2) had stronger inhibitory ability in the IgE binding assay with horsefly SGE than these wasp allergens (Vesp ma 5 and Vesp ma 2).

### Skin Prick Tests

The results of the SPTs are shown in Table S1. Of the 15 patients with wasp allergy, 11, 12, 9 and 9 (73%, 80%, 60%, and 60%) patients had positive reactions to Vesp ma 5, Vesp ma 2, Tab y 5 and Tab y 2, respectively. Of the 15 patients with horsefly allergy, 8, 10, 13 and 12 (53%, 67%, 87%, and 80%) patients had positive reactions to Vesp ma 5, Vesp ma 2, Tab y 5 and Tab y 2, respectively. The representatives of SPTs are illustrated in Figure S2.

## Discussion

In the present study, two major allergens are identified from the venoms of the wasp *V. magnifica*. They are an antigen 5 (Vesp ma 5) and a hyaluronidase (Vesp ma 2) protein, respectively.

There are more than 24 species in the wasp genus *Vespa*, but allergens have been identified thus far only in *Vespa crabro*. Hoffman and co-workers [7] reported four cases of allergic reactions to *V. crabro* stings. They found 3 of 4 patients with known reactions to *V. crabro* venom antigen 5. One patient was most reactive with hyaluronidase. Phospholipase had relatively little IgE-binding activity, although it is the major protein in this venom [7]. Few allergens have been purified or cloned from *V. crabro*. They are Vesp c 5 (Antigen 5) and Vesp c 1 (Phospholipase A_1_) [6], [7]. However, these two allergens' allergenicity was poorly understood. Thirty and 31 of 33 (91.0% and 93.9%) of subjects' sera with *Vespa* wasp allergy reacted to Vesp ma 5 and Vesp ma 2, respectively, by immunoblotting technique. Of the 15 patients with wasp *Vespa* allergy, 11 and 12 (73%, and 80%) patients had positive SPT reactions to Vesp ma 5 and Vesp ma 2 respectively. It is likely that antigen 5 and hyaluronidase are also the major allergens in *Vespa* wasp, as those from other vespid venom and horsefly. Hyaluronidases are glycoside hydrolases that cleave b-1,4-glycosidic bonds between the N-acetylglucosamine and the D-glucuronic acid of hyaluronic acid, and thereby act as spreading factors in Hymenoptera venoms [21]–[23]. Hyaluronidases have been previously recognized as major allergens in the honeybee venom, the yellow jacket venom, as well as from horsefly [21]–[23]. However, hyaluronidase has been recently shown to be only a minor vespid allergen, at least in Vespula-allergic subjects. Most of the IgE reactivity seen with hyaluronidase is directed against carbohydrates (CCDs), and binding to hyaluronidase is seen almost exclusively in wasp-honeybee double-positive patients [24], [25]. It is likely that high Ig E binding rate to Vesp ma 2 in wasp venom-allergic patients in the present study might be caused by protein epitopes as well as by CCDs.

Antigen 5 proteins belong to the CAP protein family including cysteine-rich secretory proteins, insect antigen 5 proteins, and plant pathogenesis-related proteins. Several Antigen 5 proteins from hornets, wasps, horseflies, and fire ants are identified as allergens [6], [16], [26]–[28]. They can induce acute hypersensitivity responses in humans. No native Ag 5 protein has been purified and characterized from *Vespa* wasps, and the current work is the first to prove the presence of Ag 5-like protein (Vesp ma 5) with IgE-binding ability in *Vespa* wasps.

Vesp ma 2 shares 92%, 91%, 73%, 54% and 41% sequence similarity with the Dol m 2 from *Dolichovespula maculate* [28], Ves v 2 from *Vespula vulgaris* [29], Pol a 2 from *Polistes annularies* [30], Api m 2 from *Apis mellifera* [31], and Tab y 2 from *Tabanus yao* [16], respectively. Hyaluronidases represent one of the most strongly conserved Hymenoptera allergens and are also thought to be as one of most important cross reactive allergens in yellow jacket and honeybee venom [21]. Vesp ma 5 shares 87%, 72%, 68%, 65% and 27% sequence similarity with Vesp c 5 from *Vespa crabro* [6], Dol m 5 from the *Dolichovespula maculate* [26], Ves v 5 from *Vespula vulgaris* [27], Vesp m 5 from *Vespula maculifrons* [28], and Tab y5 from *Tabanus yao* [16], respectively. Ag 5-like proteins are also strongly conserved Hymenoptera allergens. Concomitant sensitization to Hymenoptera venoms in subjects allergic to horseflies seems to be frequent, since many cases of horsefly allergy have been recorded so far [8]–[10]. Two years before, Quesrcia and co-workers [32] suggested the wasp-horsefly syndrome based on two cases of two male patients (57 and 62 years of age), who are already known as allergic to stinging hymenoptera venom. After a horsefly bite, they presented a serious 3–4 degree-type Mueller classification systemic reaction [32]. No direct proof for the wasp-horsefly syndrome is presented and no cross reactive allergen has been identified. We have identified three allergens, Tab y 1, Tab y 2 and Tab y 5 from horsefly, *Tabanus yao* Macquart [16], [17]. The current work found that these four allergens (Vesp ma 2 and Vesp ma 5, Tab y 2 and Tab y 5), can induce allergic reactions in patients with allergy to horseflies or wasps, and acted as cross reactive allergens between allergy to horseflies and wasps. Immunoblots indicated that all these four allergens bind to special IgE antibodies not only in the sera of patients with horsefly allergy but also in those of the patients with wasp allergy (Fig. 3A–D). In particular, three allergens (Vesp ma 2, Tab y 5, and Tab y 2) can simultaneously bind to their corresponding IgEs in patients with allergic reactions to horsefly bites or wasp stings, providing further evidence that they are cross reactive allergens for the horsefly-wasp syndrome (Fig. 3E). ELISA inhibition showed that they could inhibit the binding of the subjects' IgE antibodies to coated SGE or WV in a dose-dependent manner (Fig. 4). Moreover, these four cross reactive allergens can induce allergic reactions in allergic subjects by SPTs (Table 1 and Fig. S2). All these results indicate that the current four allergens are responsible for the horsefly-wasp syndrome and confirm the existence of cross reactive allergens in both horsefly and wasp. The venom hyaluronidases in hymenoptera are classical allergens made responsible for cross-reactivity. Nevertheless, their IgE reactivity is dominated by the pronounced cross-reactivity of carbohydrate determinants (CCDs) [21]. According to simple sequence analyses the Vesp ma 2 and Tab y 2 proteins contain 4 putative glycosylation sites and even the usually non-glycosylated antigen 5, Tab y 5, contains 2 putative glycosylation sites. It is highly unlikely that these sites are completely devoid of glycans. This information is potentially relevant, especially with regard to cross-reactivity. Thus, IgE reactivity is not a parameter that can be taken into account without excluding CCD-reactivities.

More than 50% of the IgE binding with the coated WV could be inhibited by the SGE (Fig. 4A and B), and more than 60% of the IgE binding with the coated SGE could be inhibited by the WV (Fig. 4C and D). The total IgE binding with the coated WV by both Tab y 5 and Tab y 2 does not exceed 45% (Fig. 4A and B), and the total IgE binding with the coated SGE by both Vesp m 5 and m 2 does not exceed 50% (Fig. 4C and D). This suggests that: (1) both the horsefly SGE and the wasp venom share a large proportion of cross reactive allergens; and (2) in addition of these four cross allergens of Vesp ma 5, Vesp ma 2, Tab y 5, and Tab y2, there are other cross reactive allergens in both the wasp and the horsefly.

In these ELISA inhibition assays, the maximal inhibition by one of these four allergens was less than 43% (Fig. 4). These results indicated that these allergens are important for the allergic reactions induced by horsefly or wasp sting but there existed other allergens in these organisms. Several lines of evidences have indicated that there are multiple allergens in the same organism. At least eight allergens were predicted to be in the saliva of *Aedes aegypti* [33]–[35]. There are more than four types of allergens in the venoms of the wasp *Polistes gallicus* [8]. In order to develop highly sensitive immunoassays for the diagnosis of horsefly or wasp allergy and effectively treat the individual who is sensitized by the particular allergen(s), further work is necessary to identify and characterize all of the allergens in these organisms.

## Supporting Information

Figure S1 **Hyaluronidase activity of Vesp ma 2.**
(TIF)

Figure S2 **Representative results of SPTs.**
(TIF)

Table S1 **Results of skin prick tests using purified allergens.**
(DOC)
