# Supplementary figures and images for: Purification and Characterization of Two New Allergens from the Venom of Vespa magnifica
Source: PLoS One. 2012 Feb 27;7(2):e31920. doi: 10.1371/journal.pone.0031920 (PMC3288059; doi:10.1371/journal.pone.0031920)

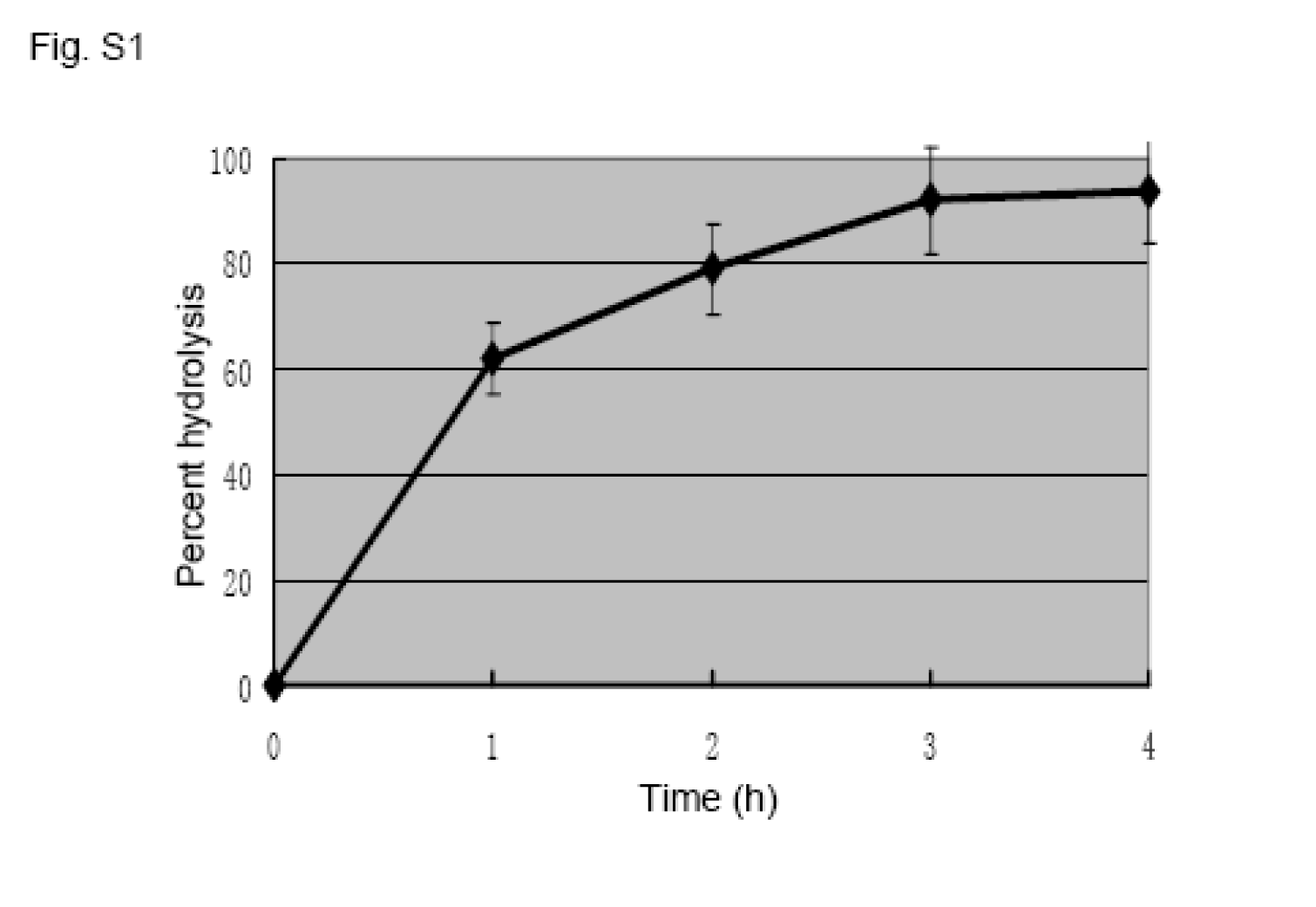

Supplement: Figure S1 — Hyaluronidase activity of Vesp ma 2. (TIF) [file pone.0031920.s001.tif]

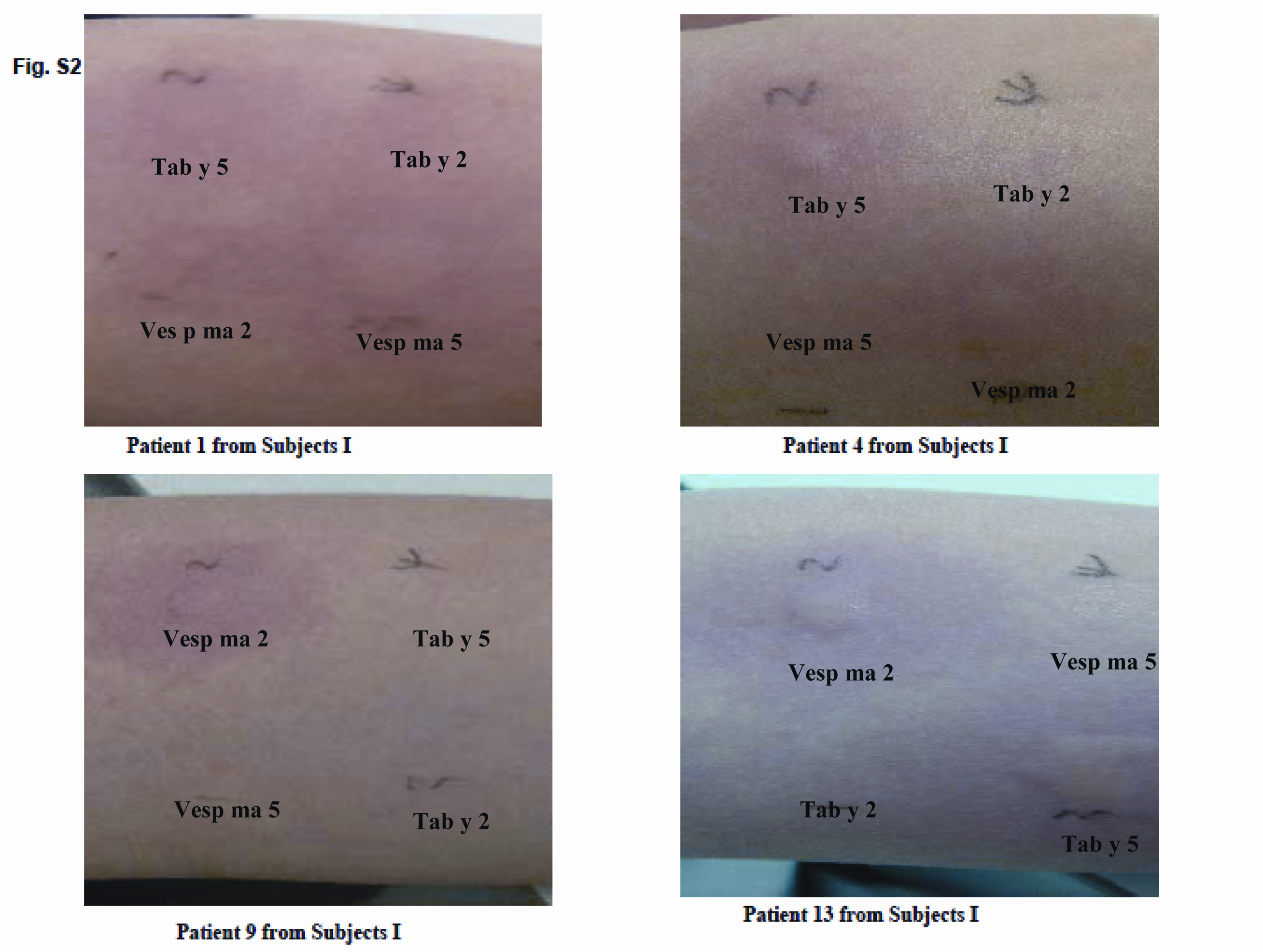

Supplement: Figure S2 — Representative results of SPTs. (TIF) [file pone.0031920.s002.tif]
