# Supplementary material for: Purification and Characterization of Two New Allergens from the Venom of Vespa magnifica
Source: PLoS One. 2012 Feb 27;7(2):e31920. doi: 10.1371/journal.pone.0031920 (PMC3288059; doi:10.1371/journal.pone.0031920)
Supplement: Table S1 — Results of skin prick tests using purified allergens. (DOC) [file pone.0031920.s003.doc]

**Table S1. Results of skin prick tests** using purified allergens

| **Subjects I** | **Net wheal size (mm)** | | | | **Subjects II** | **Net wheal size (mm)** | | | |
| --- | --- | --- | --- | --- | --- | --- | --- | --- | --- |
|  | Vesp ma 5 | Vesp ma 2 | Tab y 5 | Tab y 2 |  | Vesp ma 5 | Vesp ma 2 | Tab y 5 | Tab y 2 |
| 1 | 9.5 | 8.3 | 6.3 | 5.5 | 1 | 6.5 | 4.4 | 7.9 | 8.5 |
| 2 | 7.6 | 9.7 | <3 | 7.4 | 2 | 9.2 | 7.7 | 10.5 | 9.8 |
| 3 | 5.2 | 6.1 | 9.2 | 6.7 | 3 | <3 | <3 | 9.1 | 7.2 |
| 4 | 5.3 | 7.3 | 9.9 | <3 | 4 | <3 | 3.7 | 6.5 | <3 |
| 5 | <3 | 7.1 | <3 | <3 | 5 | 5.5 | 6.0 | 11.2 | 8.1 |
| 6 | 8.6 | 5.2 | 6.6 | 4.2 | 6 | 7.6 | <3 | <3 | 6.2 |
| 7 | 10.5 | 9.0 | 3.7 | 6.5 | 7 | 4.6 | 7.3 | 6.7 | 9.5 |
| 8 | <3 | <3 | 7.1 | 8.3 | 8 | <3 | 4.1 | 5.7 | 6.7 |
| 9 | <3 | 8.6 | <3 | <3 | 9 | <3 | <3 | 3.9 | 4.3 |
| 10 | 6.9 | 4.3 | 3.9 | <3 | 10 | <3 | 6.2 | <3 | 10.3 |
| 11 | 5.2 | 7.5 | <3 | <3 | 11 | 8.1 | 9.2 | 10.5 | 11.1 |
| 12 | 4.7 | 6.6 | 4.2 | <3 | 12 | 6.2 | <3 | 8.0 | <3 |
| 13 | <3 | 8.7 | 10.2 | <3 | 13 | <3 | 7.7 | 6.2 | 8.7 |
| 14 | 8.9 | <3 | <3 | 4.9 | 14 | 7.2 | <3 | 9.5 | <3 |
| 15 | 6.5 | <3 | <3 | <3 | 15 | <3 | 3.7 | 5.2 | 5.6 |

Subjects I: patients with wasp allergy; Subjects II: patients with horsefly allergy. Net wheal size in mm = allergen reaction size - negative control size; Negative-control wheal size: mean, 0.41 mm; median, 0 mm; range, 0-3 mm
